# Field pathogenomics and evolutionary conservation unveil CRISPR-targetable susceptibility genes for wheat blast resistance

**DOI:** 10.1038/s41598-026-36547-6

**Published:** 2026-01-18

**Authors:** Abul Khayer, Peng Ye, Fatiha Sultana Eti, Tahsin Islam Sakif, Rojana Binte Azad, Julfikar Ali, Dipali Rani Gupta, Soichiro Asuke, Qinghua Pan, Mohammad Ali Moni, Houxiang Kang, Tofazzal Islam

**Affiliations:** 1 Institute of Biotechnology and Genetic Engineering (IBGE), Gazipur Agricultural University, Gazipur, 1706 Bangladesh; 2 Institute of Plant Protection, Chinese Academy of Agricultural Sciences, Beijing, China; 3 Extension Service, WVU Plant Diagnostic Clinic, West Virginia University, Morgantown, WV 26506 USA; 4 Laboratory of Plant Pathology, Graduate School of Agricultural Science, Kobe University, Kobe, 657-8501 Japan; 5 Department of Plant Pathology, South China Agricultural University, Guangzhou, China; 6 AI & Digital Health Technology, Rural Health Research Institute, Charles Sturt University, Orange, NSW 2800 Australia; 7 Present Address: Max-Planck-Institute of Molecular Plant Physiology, Am Mühlenberg 1, 14476 Potsdam-Golm, Germany; 8 Present Address: Institute for Resistance Research and Stress Tolerance, Julius Kuehn Institute (JKI)-Federal Research Centre for Cultivated Plants, Erwin-Baur-Str. 27, 06484 Quedlinburg, Germany; 9 AI and Digital Health Technology, Artificial Intelligence and Cyber Futures Institute, Charles Sturt University, Bathurst, NSW 2795 Australia; 10 School of Information Technology, Washington University of Science and Technology, Alexandria, VA 22314 USA

**Keywords:** Wheat blast, *Magnaporthe oryzae* pathotype *Triticum*, Susceptibility genes, Durable blast resistance, Global climate change, Tendem kinase proteins, Computational biology and bioinformatics, Genetics, Microbiology, Molecular biology, Plant sciences

## Abstract

Wheat blast, caused by *Magnaporthe oryzae* pathotype *Triticum* (MoT), threatens global wheat production, yet durable resistance mechanisms remain elusive. Current strategies relying on race-specific resistance genes or fungicides are vulnerable to pathogen evolution and inefficacy. Here, we investigated field-derived transcriptomes from the 2016 Bangladesh wheat blast epidemic, a catastrophic event devastating all local varieties to identify host susceptibility (*S*) genes co-opted by MoT. By analyzing RNA-seq data from infected and healthy plants across geographically distinct regions, we pinpointed 273 consistently upregulated wheat genes, enriched in defense-related pathways. Ortholog analysis with rice, a model for blast resistance, identified three conserved susceptibility (*S*)-gene candidates: *TaSULTR3-3B* (an ortholog of a rice bacterial blight susceptibility gene), *TaSTP3-4D* (associated with stripe rust), and *TaMLO1-5A* (a wheat powdery mildew susceptibility gene). While all three candidates exhibited significant expression correlation with *M. oryzae Triticum* (MoT) effectors in field-derived samples, in planta spike assays revealed distinct expression dynamics. Only *TaMLO1-5A* was significantly upregulated in the susceptible cultivar BARI Gom 26 following MoT inoculation, with no induction observed in the resistant cultivar S-615 (carrying *Rmg8*). Conversely, *TaSULTR3-3B* and *TaSTP3-4D* did not show significant induction under the specific conditions and time points of the in planta spike assays. This discrepancy potentially arises from tissue-specific regulation (spike vs. leaf), environmental variations, or differences in sampling time points between the field and greenhouse experiments. Disruption of such *S* genes, validated in other cereals for durable resistance, offers a transformative strategy to engineer non-race-specific wheat blast resilience. Our findings shift the paradigm from transient resistance genes to foundational susceptibility networks, proposing CRISPR-based editing of the candidate gene as an actionable target. This approach, resilient to pathogen evolution, could preempt epidemics in climate-vulnerable regions, safeguarding global wheat security. By bridging field pathogenomics and evolutionary genomics, we provide a roadmap for sustainable disease management in an era of expanding fungal threats.

**Supplementary Information:**

The online version contains supplementary material available at 10.1038/s41598-026-36547-6.

## Introduction

Wheat (*Triticum aestivum* L.), a crop vital to global food security, faces an escalating threat from wheat blast, a devastating disease caused by the fungal pathogen *Magnaporthe oryzae* pathotype *Triticum* (MoT). First documented in Brazil in 1985 after a host jump from ryegrass facilitated by mutations in the fungal effector *PWT3*, this disease has since triggered recurrent epidemics across South America, devastating yields in Argentina, Bolivia, and Paraguay. Its recent transcontinental spread to Bangladesh (2016) and Zambia (2018) via a clonal South American lineage has intensified fears for food security in Asia and Africa, where wheat sustains millions^1–4^. Disturbingly, a sporadic detection in a single wheat spike in an experimental field in the U.S. (2011) and recent detections of potential wheat infecting strains in the grasses in Germany signal capacity wheat blast fungus to infiltrate major wheat-producing zones, including Europe’s cereal belt^4,5^. Under conducive conditions, wheat blast causes yield losses of up to 100% within weeks, decimating entire harvests. With Bangladesh now an epidemiological hotspot, neighboring nations like India and China, collectively producing approx. 35% of global wheat, face existential risks to their grain systems^6^.

Current mitigation strategies remain critically inadequate. Fungicides, often deployed post-symptomatically, falter against MoT’s rapid colonization of and blocking this vascular tissue in wheat spike, while traditional resistance breeding relies on race-specific nucleotide-binding leucine-rich repeat (NLR) proteins (*R* genes) that pathogens rapidly evade via effector mutations^7^. Although 11 MoT-specific *R* genes have been identified, their utility is hampered by temperature sensitivity, developmental stage restrictions, and ephemeral efficacy^8,9^. These limitations underscore the urgency of non-race-specific, evolutionarily robust resistance mechanisms.

A paradigm-shifting alternative is to target susceptibility (*S*) genes, which are host factors that pathogens co-opt to enable infection. Unlike *R* genes, *S*-gene-mediated resistance is genetically recessive, non-race-specific, and durable, as demonstrated by *mlo*-based resistance to powdery mildew in wheat and barley. By disrupting *S* genes, which encode critical infection checkpoints, breeders impose a higher evolutionary barrier on pathogens compared to NLR-effector arms races^10,11^. For instance, CRISPR editing of the *MLO* locus in wheat conferred robust powdery mildew resistance without yield penalties^12^. Despite this promise, no *S* genes conferring wheat blast resistance have been identified, leaving a critical gap in durable disease management.

The 2016 Bangladesh epidemic, which uniformly devastated all local wheat varieties (carried no known *R* genes), provides a unique opportunity to dissect MoT’s host dependencies. Using field pathogenomics, an approach combining in situ transcriptomics and pathogen tracking, we previously traced the outbreak to a clonal South American MoT lineage. Here, we leveraged unpublished RNA-seq data of wheat from these pandemic fields, accessible *via* an open wheat blast database (http://openwheatblast.net/), to test the hypothesis that MoT’s uniform destruction of diverse wheat varieties reflects exploitation of conserved host *S* genes^13^. Through integrative analysis of field-derived transcriptomes, the objectives of our study were to (i) identify wheat genes consistently upregulated during natural MoT infection; (ii) construct host-pathogen interaction networks linking fungal effectors to co-expressed wheat transcripts; (iii) prioritize candidates via evolutionary conservation with rice blast-associated *S* genes; and (iv) validate susceptibility roles through in silico functional annotation and also via an in planta experiment. We captured gene expression dynamics reflective of real-world pathogen pressure by analyzing field epidemics rather than artificial lab infections. Our approach bridges evolutionary genomics by exploiting rice’s well-characterized blast resistance mechanisms for unveiling CRISPR-targetable susceptibility networks in wheat. We hypothesized *TaSTP3-4D* (stripe rust susceptibility), *TaMLO1-5A* (powdery mildew susceptibility), and *TaSULTR3-3B* (bacterial blight susceptibility in rice) as candidate *S* genes that not only elucidate MoT’s infection strategy but also deliver actionable targets for genome editing using the CRISPR-Cas system. This strategy, validated in wheat and other cereals for durable resistance, could preempt outbreaks in climate-vulnerable regions, safeguarding global wheat production against an evolving fungal threat.

## Materials and methods

### Transcriptome sequencing of collected samples of wheat and blast from the pandemic field of Bangladesh

Symptomatic (blast-infected) and asymptomatic leaf blades of wheat were sampled from the same pandemic-afflicted wheat fields across two geographically distinct regions in Bangladesh, characterized by differing climatic conditions (Table 1). Total 16 samples from four wheat cultivars with 2 biological replicates were included to capture host-specific responses. The disease severity in the wheat field, leaf sample collection, and climate descriptions about the two districts were described at Islam et al.^2^. Immediately after collection, both infected and healthy leaf tissues were sliced into 0.5 × 1.0 cm strips and flash-preserved in 1 mL RNAlater™ RNA Stabilization Solution (Thermo Fisher Scientific, Basingstoke, UK) to arrest RNA degradation. Total RNA was isolated using the RNeasy Plant Mini Kit (Qiagen, Manchester, UK), with quantity and integrity assessed via the Agilent 2100 Bioanalyzer (Agilent Technologies, Edinburgh, UK). High-quality RNA (RIN ≥ 8.0) was used to construct cDNA libraries with the Illumina TruSeq RNA Sample Preparation Kit (Illumina, Cambridge, UK), followed by library quality validation on the same Bioanalyzer platform^2^. Paired-end sequencing (101-bp reads) was performed on an Illumina HiSeq 2500 system at The Genome Analysis Centre (TGAC), UK. The quality of the data was described at Islam et al.^2^. While pathogen-derived transcriptomic data from these samples had previously been analyzed to trace the outbreak’s origin^2^, the corresponding wheat host transcriptomes remained unexplored until this study. RNAseq data used in this study are available and taken from open wheat blast website (http://openwheatblast.net/).

**Table 1:** Description of wheat samples collected from the pandemic field of Bangladesh.

Accessions	Location	Cultivar	Reaction	Sample groups
ERR1360178	Meherpur	BARI Gom 25	Asymptomatic	F2-0
ERR1360179	Meherpur	BARI Gom 25	Asymptomatic	F2-0
ERR1360180	Meherpur	BARI Gom 26	Asymptomatic	F5-0
ERR1360181	Meherpur	BARI Gom 26	Asymptomatic	F5-0
ERR1360182	Jhinaidaha	Shotabdi	Asymptomatic	F7-0
ERR1360183	Jhinaidaha	Shotabdi	Asymptomatic	F7-0
ERR1360184	Jhinaidaha	Kanchan	Asymptomatic	F12-0
ERR1360185	Jhinaidaha	Kanchan	Asymptomatic	F12-0
ERR1360186	Meherpur	BARI Gom 25	Symptomatic	F2-1
ERR1360187	Meherpur	BARI Gom 25	Symptomatic	F2-1
ERR1360188	Meherpur	BARI Gom 26	Symptomatic	F5-1
ERR1360189	Meherpur	BARI Gom 26	Symptomatic	F5-1
ERR1360190	Jhinaidaha	Shotabdi	Symptomatic	F7-1
ERR1360191	Jhinaidaha	Shotabdi	Symptomatic	F7-1
ERR1360192	Jhinaidaha	Kanchan	Symptomatic	F12-1
ERR1360193	Jhinaidaha	Kanchan	Symptomatic	F12-1

### Mapping raw short reads

**Fig. 1:**
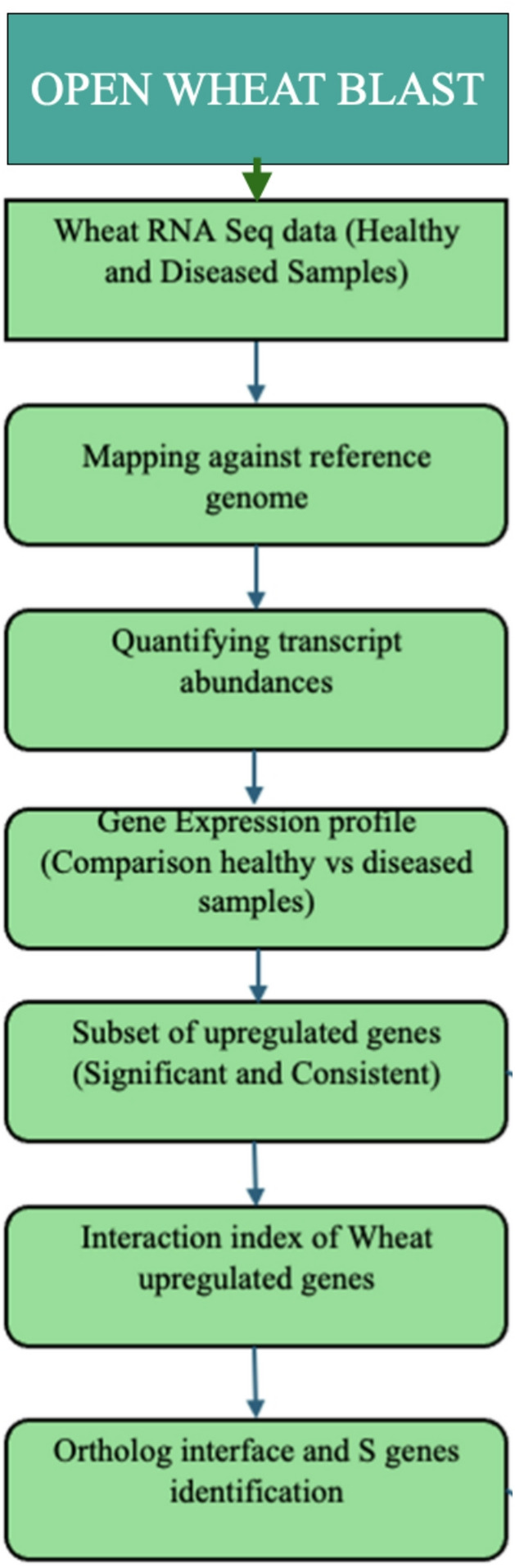
Key steps of the RNA-seq data processing of wheat samples.

Raw RNA-seq reads from wheat were aligned to the *Triticum aestivum* Chinese Spring coding sequences (IWGSC RefSeq v2.1), retrieved from Ensembl Plants and pathogen-derived reads were aligned separately to the coding sequences of the wheat blast strain *BR32* (available via FungiDB) using Kallisto (v0.48.0)^14^, a pseudo aligner optimized for rapid and accurate transcript quantification. Default Kallisto parameters were applied, including sequence-based bias correction and bootstrap to quantify transcript abundance uncertainty. This dual-mapping strategy enabled simultaneous analysis of host and pathogen gene expression profiles from the same infected tissues (Fig. 1).

### Transcript quantification and abundance estimation

Post-mapping quantification revealed 133,346 wheat transcripts and 14,349 MoT transcripts across all samples. Kallisto-generated abundance files^14^ provided transcript-level metrics, including transcript ID, length, effective length, raw read counts, and TPM (transcripts per million) values, with TPM enabling cross-sample normalization for expression comparisons. To streamline downstream analyses (e.g., differential expression, co-expression network modeling), transcript IDs, raw counts, and TPM values were consolidated into a unified tab-separated file^15^, structured with rows representing transcripts and columns corresponding to individual samples. This standardized matrix facilitated systematic interrogation of host-pathogen transcriptome dynamics during infection.

### Identification and analysis of differentially expressed genes (DEGs)

To characterize the transcriptional response of wheat to MoT infection, differential gene expression analysis was performed using the edgeR package (v3.40.2) in R^16^. Raw transcript counts from healthy (control) and blast-infected samples were normalized via the trimmed mean of M-values (TMM) method to correct for library size differences and compositional bias. A generalized linear model (GLM) framework was applied to estimate dispersions, accounting for biological variability across replicates and experimental conditions (e.g., genotype, geographical location). DEGs were identified through likelihood ratio tests comparing infected and control groups, with statistical significance determined using the exact test function in edgeR. Log_2_ fold change (log_2_FC) values quantified expression differences, and false discovery rate (FDR) correction (Benjamini-Hochberg method) was applied to adjust for multiple testing. Genes with FDR < 0.01 and |log_2_FC| > 1 were classified as differentially expressed, yielding distinct sets of upregulated (log_2_FC > 1) and downregulated (log_2_FC < − 1) transcripts. Expression patterns of DEGs were visualized using clustering (Euclidean method in both rows and columns) heatmaps generated with the ComplexHeatmap package (v2.13.1). To know shared and unique DEGs across genotypes and locations, an UpSet plot was generated using the UpSetR package (v1.4.0), highlighting intersections of upregulated gene sets.

### Pathogen transcript quantification

Parallel analysis of MoT transcripts isolated from infected tissues was conducted using identical statistical thresholds. This dual approach enabled systematic exploration of host-pathogen transcriptional interplay during infection.

### Gene ontology (GO) enrichment analysis of wheat transcripts

To identify biological processes, molecular functions, and cellular components underpinning wheat’s response to MoT infection, Gene Ontology (GO) enrichment analysis was performed on DEGs using ShinyGO v0.77. The study employed the wheat GO annotation database (GO Release 2023-08) as a reference, with significance thresholds set at FDR < 0.05 and fold enrichment > 2. Enriched GO terms were ranked by hypergeometric test p-values, adjusted for multiple testing using the Benjamini-Hochberg method. Results were visualized as a scatter plot generated within ShinyGO, where node size represented the number of enriched genes per term, color intensity denoted the significance level (-log_10_(FDR)).

### Ortholog analysis and evolutionary context

We conducted an ortholog analysis to enhance our understanding of the evolutionary context and reinforce our confidence in the functions of the selected wheat genes. We focused on comparing these wheat genes with those in the rice genome, as rice serves as a well-established model organism for studying blast infections. This comparative approach enabled us to identify orthologous genes in rice that may share functional similarities with our wheat gene set. The ortholog analysis was carried out using the g: Profiler platform (https://biit.cs.ut.ee/gprofiler/orth), which provided critical insights into the conservation of gene functions between these two agriculturally significant cereal crops. We created a Sankey plot using R programming to visualize the relationships among the identified orthologs. This visualization effectively illustrates the connections and similarities between the wheat and rice genomes, enhancing our understanding of the evolutionary context of the selected wheat genes.

### Fungal inoculum preparation for in planta study

A highly virulent strain of MoT, BTJP 4–5, was used for plant inoculation. The blast fungus was grown on Petri dishes containing potato dextrose agar (PDA) medium and incubated for 7 days at 25 °C (Gupta et al., 2020). The 7-day-old culture plates were flooded with 5 mL of sterilized water, and aerial parts of the fungal colony were dislodged by gently rubbing the surface with a sterilized paintbrush. The cultures were then exposed to fluorescent light for 3 days at 25 °C to induce sporulation^17^. Spores were harvested using a sterilized brush and suspended in sterilized water containing 0.01% (v/v) Tween 20. The spore suspension was filtered through cheesecloth, and the spore density was adjusted to 1–1.5 × 10^5^ conidia/mL using a Neubauer hemocytometer.

### *In planta* transcriptomic experiment

Wheat spikes at the flowering stage were selected and then inoculated with the conidia MoT isolate BTJP 4–5 for RNA sequencing analysis. Immediately following inoculation, each spike was covered with a plastic bag moistened on the inside to create a high-humidity microenvironment (~ 99%) for 48 h. After removing the bags, the plants were placed in a growth chamber at 25–28 °C with a 16-hour light cycle. Two wheat genotypes were used: a susceptible cultivar, BARI Gom 26 and a wheat genotype, S-615 carrying a major wheat blast resistance gene, *Rmg8*. Samples were collected at 96 h post-inoculation (hpi), with two biological replicates processed per treatment. Mock controls were treated with Tween 20 only. Total RNA was extracted using the Total RNA Extraction Kit (Sangon Biotech, B511321), following the manufacturer’s instructions. Sequencing was performed on the Illumina NovaSeq 6000 platform with paired-end reads (2 × 150 bp), generating ~ 30 million reads per sample. Raw reads were quality-checked with FastQC, and summary reports were generated using MultiQC. Adapters and low-quality sequences were removed using Trim Galore v0.6.10. Cleaned reads were aligned to the wheat reference genome (*Triticum aestivum*, IWGSC RefSeq v2.1) using BWA-MEM v0.7.17. Transcript assembly and gene expression quantification were performed using StringTie.

## Results

### Wheat gene expressions to blast infection

**Fig. 2:**
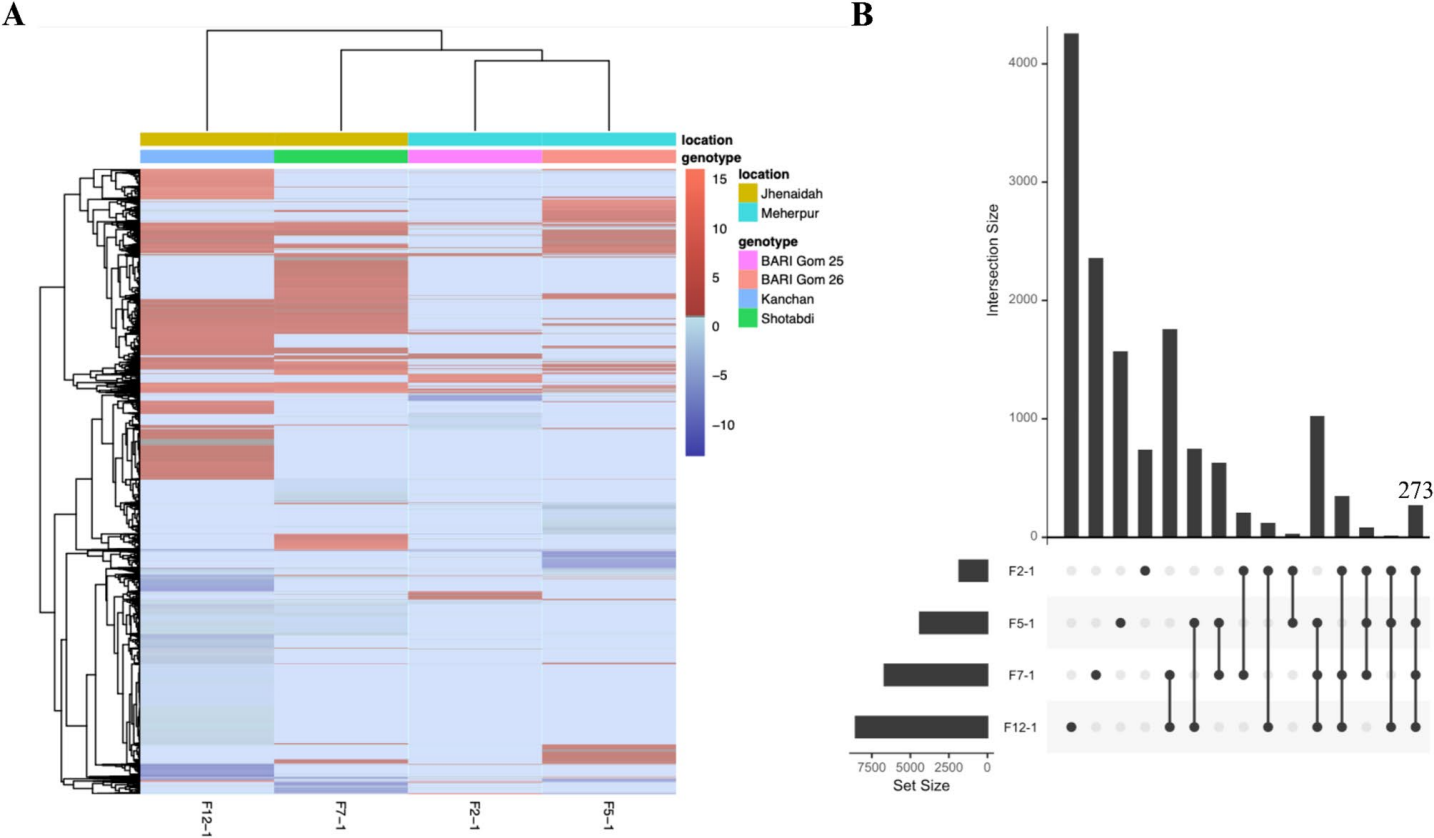
Differential gene expression profile of wheat genes in response to blast disease across two locations in Bangladesh. (**A**) The heatmap displays the log₂FoldChange values of differentially expressed genes (dark red means upregulation and blue means downregulation) in diseased wheat samples compared to their respective healthy controls. Gene expression patterns were analyzed to identify transcriptional changes associated with blast disease infection across multiple geographical locations. The log₂FoldChange values represent the relative expression levels in infected samples normalized to corresponding healthy samples. Genes with |log₂FoldChange| > 1 and FDR < 0.01 were considered differentially expressed, indicating their potential role in blast disease response. Clustering of genes highlights common and location-specific gene expression patterns. (**B**) The figure displays the total number of upregulated genes in each sample. Vertical bars represent the intersection size of genes, each shared between samples, while horizontal bars indicate the total gene size of each sample. The dots connecting the samples denote the size of the gene set specific to the respective samples. Genes with log₂FoldChange > 1 and FDR < 0.01 were considered upregulated.

To investigate the generic genetic response of wheat to the blast fungus, we conducted a comprehensive analysis of transcriptomic variations in wheat samples collected from Jhenaidah and Meherpur. Our comparison of gene expression profiles revealed significant differences between the two locations, with Jhenaidah exhibiting more upregulated genes than Meherpur (Fig. 2A). We further explored the transcriptomes of wheat cultivars to identify distinct gene clusters with cultivar-specific expression patterns (Fig. 2A). Importantly, we identified a gene cluster consisting of 273 genes that consistently showed upregulation in response to blast infection in both Jhenaidah and Meherpur and across all wheat varieties (Fig. 2B). This location-genotype specific and consistent cluster of upregulated genes showed us the complex nature of the transcriptome response of wheat to blast.

### Defense-related functions are largely enriched in consistently upregulated wheat genes

**Fig. 3:**
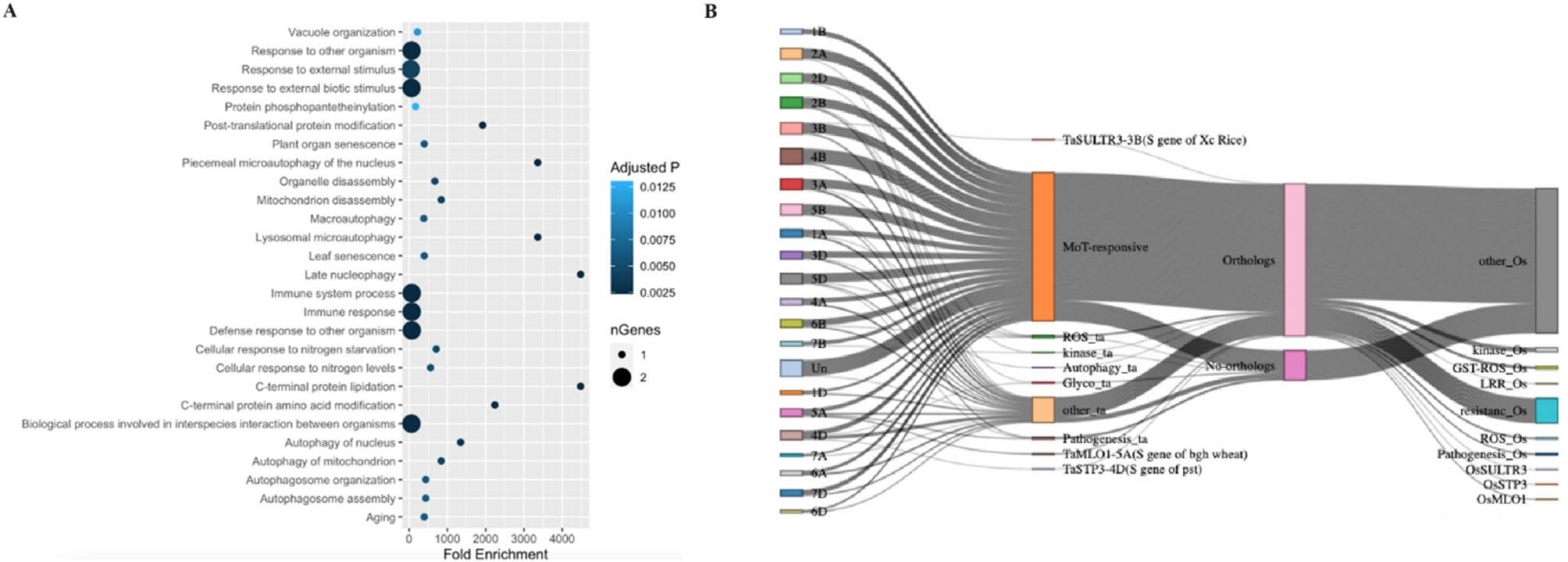
Gene ontology enrichment analysis and evolutionary aspect for the commonly upregulated genes. (**A**) Y-axis shows the ontology terms and X-axis shows how significantly enriched those terms are with the genes. The color of the points on the plots denotes the corrected P-values and size of the points indicates the number of genes linked to the ontology terms. (**B**) Orthologue’s intersection of commonly upregulated genes with Rice. Left-sided color bars denote wheat chromosomes, and each of the threads is one of the genes respective to the chromosomes. The next color bars from left denote the description/putative functions of the respective genes. The next color bars show if the respective genes are orthologous to Rice. Bars on the right side show the description of orthologous Rice genes.

To explore the biological process of the consistently upregulated genes across all analyzed samples, we conducted a gene ontology (GO) enrichment analysis (Fig. 3A). This analysis identified a strong association between the upregulated genes and several specific ontological categories. Among the enriched categories, terms associated with autophagy, defense response to other organisms, plant organ senescence, response to other organisms as well as biotic stimulus were notably prominent. Furthermore, our analysis demonstrated a significant correlation between the upregulated genes and immune system activation pathways (Fig. 3A). The GO analysis showed that the 273 consistently upregulated genes have defense/immunity related putative functions.

### Converseness of consistently upregulated genes reveal *S* genes of wheat.

**Fig. 4:**
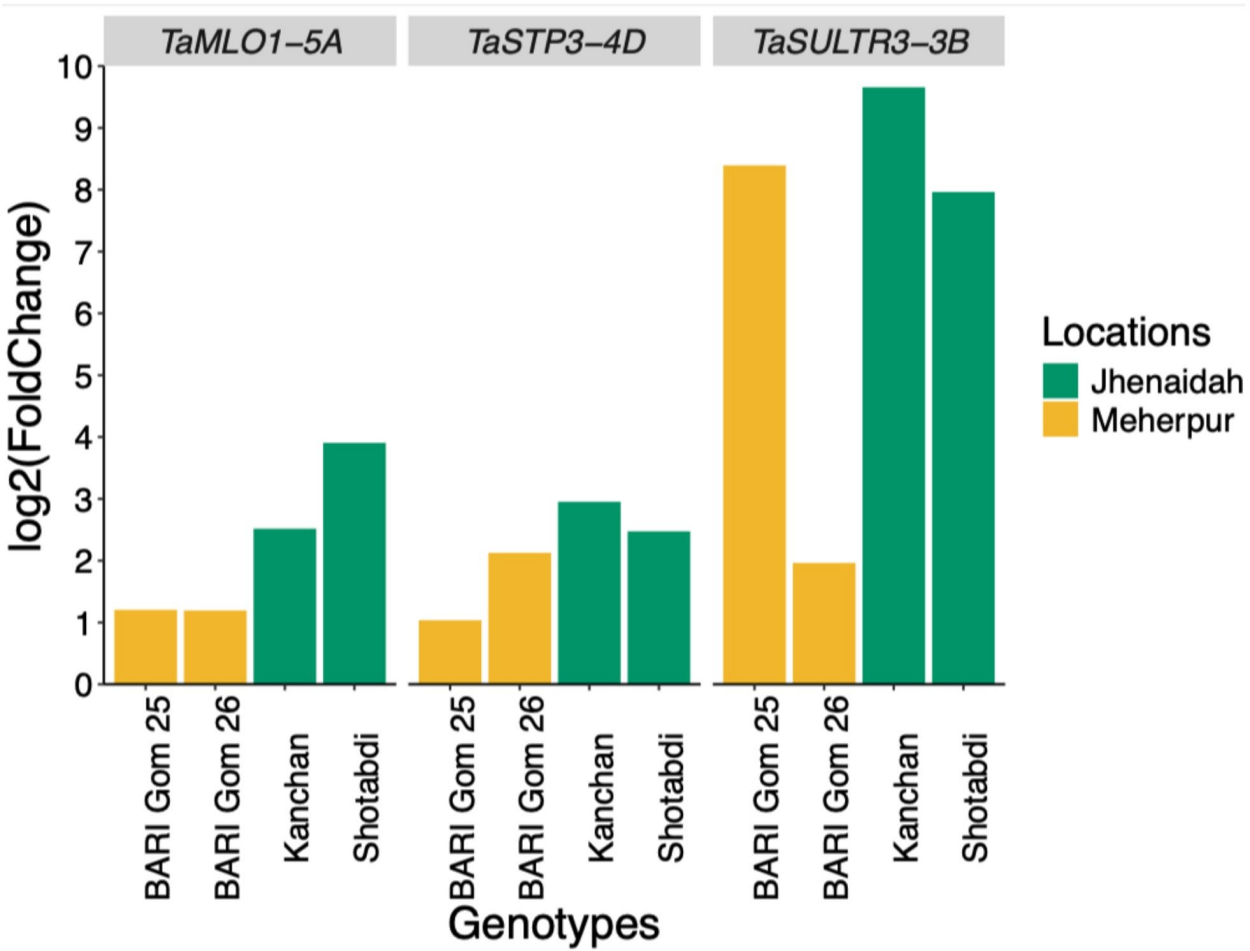
Differential expression of the three *S* genes in response to blast disease across two different locations in Bangladesh in four genotypes.

To know the conserveness, we then focused on the relationships of the 273 consistently upregulated genes in wheat with genes in rice, a well-established model organism for studying blast disease. Notably, of the 273 upregulated wheat genes, 66 exhibited a unique characteristic: they lack identifiable orthologs in rice (Fig. 3B). We found three genes which are previously associated with susceptibility to major pathogens of wheat and rice. One is *TaSULTR3-3B*, identified as an ortholog that contributes to susceptibility to bacterial blight pathogen (*Xanthomonas oryzae* pv. *oryzae*) of rice. Additionally, *TaMLO1-5A*, a gene linked to susceptibility to the powdery mildew pathogen (*Blumeria graminis* f. sp. *tritici*) of wheat, was also included. Furthermore, the identification of the *TaSTP3-4D*, which is associated with susceptibility to the stripe rust pathogen (*Puccinia striiformis* f. sp. *tritici*) of wheat (Table 2). The differential expression of *TaMLO1-5A* was higher in all locations and most of the genotypes whereas *TaSULTR3-3B* and *TaSTP3-4D* had relatively lower expression in both locations (Fig. 4). We generated an expression-correlation network between wheat *S* genes and highly expressed MoT genes, thereby finding two of the *S* genes showed high edges with MoT highly expressed genes (Supplementary Fig. 1).

**Table 2:** Description of susceptibility genes reported for related pathogens of blast in wheat and rice.

S gene	IDs	Description	Disease	Crop
*TaSULTR3-3B*	*TraesCS3B02G323500.2*	Ortholog of a *S* gene	Bacterial blight	Rice^18^
*TaMLO1-5A*	*TraesCS5A02G494800.1*	*S* gene	Powdery mildew	Wheat^12^
*TaSTP3-4D*	*TraesCS4D02G243100.1*	*S* gene	Stripe rust	Wheat^19^

The graph displays the log₂FoldChange values of differential expression of the *S* genes (strips) in diseased wheat samples compared to their respective healthy controls in the four genotypes (x-axis). The expression patterns were analyzed to identify transcriptional changes associated with blast disease infection across two geographical locations (color grouped per strip). The log₂FoldChange values represent the expression levels in infected samples compared to corresponding healthy samples. Genes with |log₂FoldChange| > 1 and FDR < 0.01 were considered differentially expressed, indicating their potential role in blast disease response.

### In planta experiment confirmed *TaMLO1-5A*, as a *S* gene of wheat-blast

**Fig. 5:**
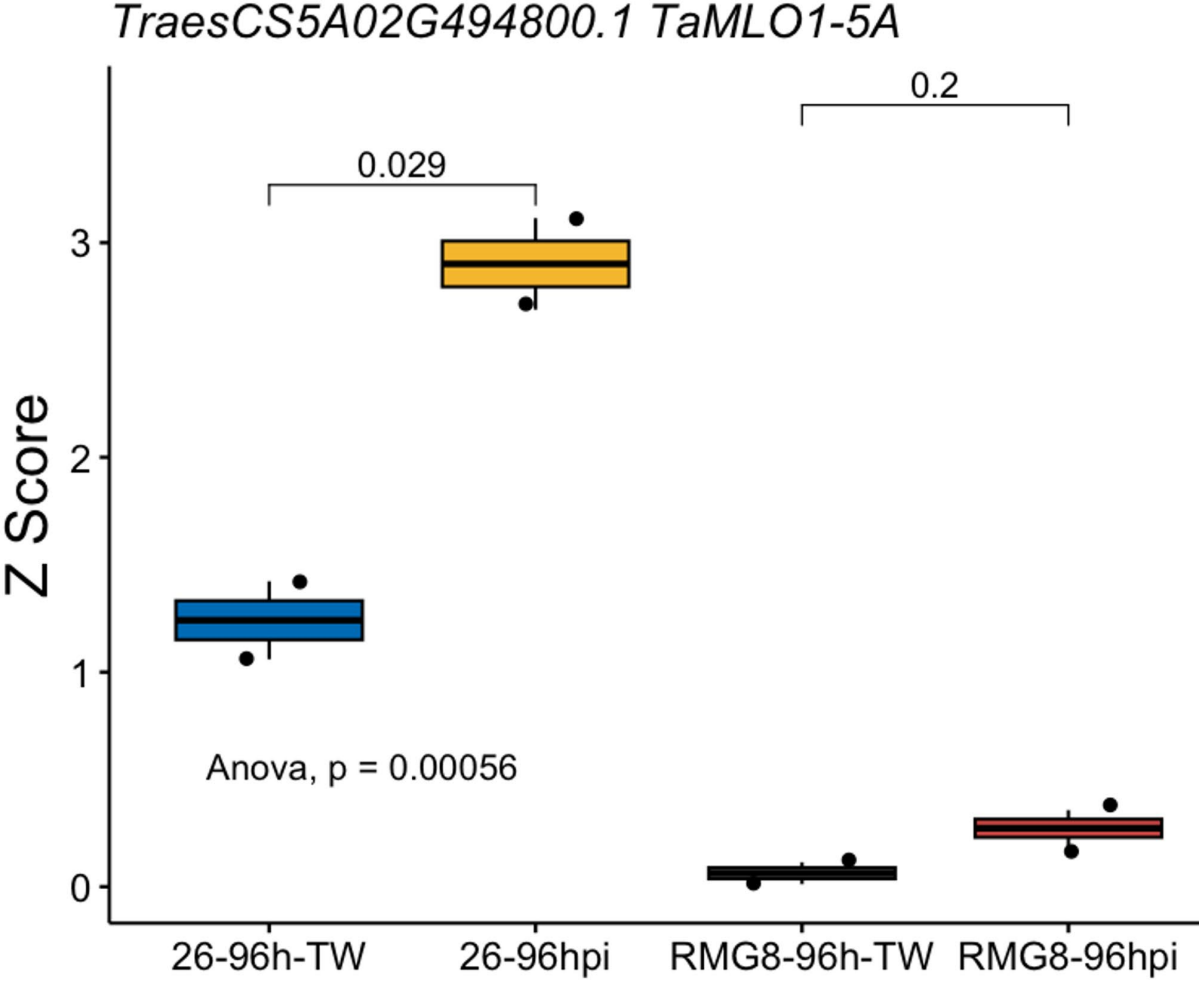
*TaMLO1-5A* expression in susceptible (BARI Gom 26) and resistant wheat samples.

To investigate gene expression differences between wheat genotypes with contrasting responses to wheat blast, we conducted an in planta experiment using two genotypes: BARI Gom 26 (a susceptible variety which was devastated by wheat blast in 2016 Islam et al.^2^ and a resistant genotype known to carry a cloned wheat blast resistance gene, *Rmg8*^20^. Both genotypes were challenged with MoT, alongside their respective non-inoculated controls. RNA sequencing was performed on all samples. Among the three *S* genes analyzed, *TaMLO1-5A* showed a significant upregulation in BARI Gom 26 following MoT inoculation (26–96 h post-inoculation, Fig. 5), compared to its control. In contrast, this gene did not show significant expression changes in the resistant genotype upon MoT infection. *TaSULTR3*-*3B* and *TaSTP3*-*4D* were not significantly induced in the comparisons under the conditions and time points after artificial inoculation.

The boxplot illustrates the normalized expression (Z-score) of the susceptibility gene *TaMLO1-5A* in MoT-inoculated and control samples from two wheat genotypes: BARI Gom 26 (26), a susceptible variety, and a resistant genotype carrying a cloned wheat-blast resistance gene, *Rmg8* in S615 genotype (RMG8). The Y-axis represents Z-score values, allowing for standardized comparison across samples, while the X-axis shows the different sample groups. “26–96 h-TW” refers to BARI-GOM-26 samples not inoculated with MoT, and “26–96hpi” refers to the same genotype inoculated with MoT. Similarly, “RMG8–96 h-TW” indicates non-inoculated resistant genotype’s samples, and “RMG8–96hpi” indicates MoT-inoculated resistant genotype’s samples. A t-test was used to compare the treated samples with their respective controls, and statistically significant differences (*p* < 0.05) are marked above the corresponding boxes, with square brackets highlighting the compared groups. An overall ANOVA was also performed to determine if expression differences across all groups were statistically significant (*p* < 0.05).

## Discussion

In the current study, we utilized field pathogenomics and evolutionary conservation to redefine the battle against wheat blast, and identified *TaSULTR3-3B*, *TaSTP3-4D*, and *TaMLO1-5A* as susceptibility (*S*) genes that could underpin *M. oryzae Triticum* (MoT) infection. By analyzing RNA-seq data from the 2016 Bangladesh epidemic, a natural laboratory where all local wheat varieties succumbed uniformly to blast, we circumvented the artificiality of lab-based infection models, capturing gene expression dynamics under real-world pathogen pressure. This dual approach, field-driven discovery coupled with cross-species genomic insights, provides a robust framework to identify *S* genes with translational potential.

A hallmark of our finding is the convergence of *TaMLO1-5A* (a powdery mildew susceptibility ortholog) and *TaSTP3-4D* (linked to stripe rust) as facilitators of wheat blast, which highlights a mechanistic overlap between fungal pathogens that exploit common host vulnerabilities. Such parallels mirror findings in rice, where *Xa13* alleles mediate susceptibility to both bacterial blight and fungal pathogens^20,21^, suggesting that *S* genes may act as hubs for multiple disease interactions. Plants transport sugars via sugar transporters, and pathogens hijack or use their own transport to redistribute plant sugar to benefit infection in plants^22^. Disrupting these hubs through CRISPR editing could confer broad-spectrum resistance, a critical advantage in an era of climate-driven pathogen range expansion. For example, expression of three rice sucrose transporter genes, *SWEET11*, *SWEET12*, and *SWEET14* is required for bacterial blight disease susceptibility. Editing of these three host *S*-genes result in rice lines with robust, broad-spectrum resistance against the bacterial blight pathogen, *Xanthomonas oryzae* pv. *oryzae*^23^. Unlike race-specific *R* genes, which pathogens rapidly evade via effector mutations, *S*-gene disruption erases molecular footholds critical for infection, imposing a higher evolutionary barrier. This strategy aligns with successes in barley (*mlo*) and tomato (*eIF4E*), where recessive *S*-gene knockouts have delivered decades of durable resistance. *S*-gene disruption may cause trade-off with other phenotype such as yield but with precise editing of the genes could lead to resistance without yield penalty^11,23–26^. Recent studies showed that genes act as multiple pathogens responsive in wheat^27–29^.

Although our findings underscore the promise of *S*-gene editing, they also highlight technical and biological challenges. The pleiotropic roles of *S* genes, such as *TaSTP3-4D*’s involvement in hexose transport, necessitate tissue-specific targeting to avoid compromising plant fitness. Advances in prime editing and tissue-specific promoters could mitigate these risks, enabling precise disruption of susceptibility pathways in floral tissues, the primary site of MoT colonization^19,30,31^. Furthermore, the clonal dominance of South American MoT lineages suggests that *S*-gene editing may face fewer evolutionary counteradaptations compared to *R*-gene strategies, though vigilance against pathogen plasticity remains essential.

To validate the field observations, we conducted a controlled in planta experiment in a greenhouse setting using two contrasting wheat genotypes, a susceptible wheat cultivar, BARI Gom 26, which exhibited severe yield losses under field conditions during the wheat blast outbreak in 2016, and a resistant genotype, S-615 carrying the *Rmg8* resistance gene^32,33^. The aim was to assess the expression dynamics of selected *S* genes following inoculation with the wheat blast pathogen MoT. Our results revealed that *TaMLO1-5A*, one of the candidate *S* genes, was significantly upregulated in BARI Gom 26 upon pathogen inoculation compared to its mock (Fig. 5). In contrast, no statistically significant difference in *TaMLO1-5A* expression was observed between inoculated and mock samples of the resistant *Rmg8*-carrying genotype. This suggests a potential role of *TaMLO1-5A* in blast susceptibility, particularly in the context of genotypes lacking effective resistance alleles. However, the other two candidate *S* genes, *TaSULTR3-3B* and *TaSTP3-4D*, were not found in the *in planta* assays under conditions and time points. A plausible explanation for this absence is the difference in the tissue type sampled, environmental conditions and time points of sampling after pathogen get in contact with hosts between the field and greenhouse experiments. In the field, RNA was extracted from leaf tissues, as spike tissues were largely decimated by the pathogen. However, in the greenhouse experiment, spike tissues were used for RNA extraction. Given that *TaSULTR3-3B* and *TaSTP3-4D* encode transport proteins likely involved in nutrient mobilization, their expression may be spatially regulated and preferentially localized to foliar tissues or induced at later stages of infection^34,35^. These findings highlight the tissue-specific and genotype-dependent expression patterns of *S* genes in wheat under blast infection and underscore the importance of sampling strategy and infection stage when interpreting transcriptional responses.

Our study’s reliance on *in planta* replicated experimental validation, while expedient for prioritizing candidates, underscores the need for field experimental confirmation using both leaf and spike tissues under varying time points and environmental conditions. For instance, *TaSULTR3-3B*’s role in wheat blast susceptibility, inferred from rice orthologs linked to bacterial blight^26,36,37^, requires functional validation through knockout assays. Additionally, field-derived transcriptomes, though ecologically relevant, may overlook genes expressed during early infection stages or in root tissues, potentially missing key susceptibility factors. Future work must integrate spatial-temporal transcriptomics and proteomics to map host-pathogen interactions comprehensively. The deployment of CRISPR-edited wheat lines in climate-vulnerable regions like Brazil, Bangladesh and Zambia represents a critical next step. These hotspots, where MoT’s spread threaten to destabilize food security^37–40^, offer ideal testing grounds to assess resistance durability under diverse environmental stresses^40,41^. Although the expression of certain tandem kinase proteins (TKPs), such as those encoded by *Rmg8* and *Rwt4*, activates host defenses upon recognizing specific effectors^42,43^, their utility in breeding programs remains limited to green house. Engineering these TKPs into durable blast-resistant wheat varieties is challenging due to their temperature sensitivity and the race-specific nature of the *M. oryzae Triticum* (MoT) fungus. Consequently, precise editing of susceptibility (*S*) genes in wheat offers a more promising strategy for developing broad-spectrum and stable resistance against MoT^12^. Beyond wheat, our network-driven approach combining field pathogenomics with evolutionary conservation could accelerate *S*-gene discovery in other crops, from rice to maize, fostering a new era of non-race-specific resistance. As rising temperatures expand MoT’s reach into temperate zones^40–42^, proactive CRISPR-Cas editing of putative MoT susceptibility gene hubs may preempt pandemics, transforming crop protection from reactive to predictive. By shifting the paradigm from ephemeral *R* genes to foundational *S*-gene networks, this work advances wheat blast resistance and charts a roadmap for sustainable disease management in the face of evolving fungal threats.

## Supplementary Information

Below is the link to the electronic supplementary material.

Supplementary Material 1

Supplementary Material 2

## Data Availability

The transcriptomics data for both wheat and blast can be freely accessed on Open Wheat Blast (http://openwheatblast.net/raw-data/). Data that support the findings of this study are available within the paper and its supplementary materials.
