# Supplementary material for: Field pathogenomics and evolutionary conservation unveil CRISPR-targetable susceptibility genes for wheat blast resistance
Source: Sci Rep. 2026 Jan 18;16:5677. doi: 10.1038/s41598-026-36547-6 (PMC12891608; doi:10.1038/s41598-026-36547-6)
Supplement: Supplementary file 2 — Supplementary Material 2 [file 41598_2026_36547_MOESM2_ESM.pdf]

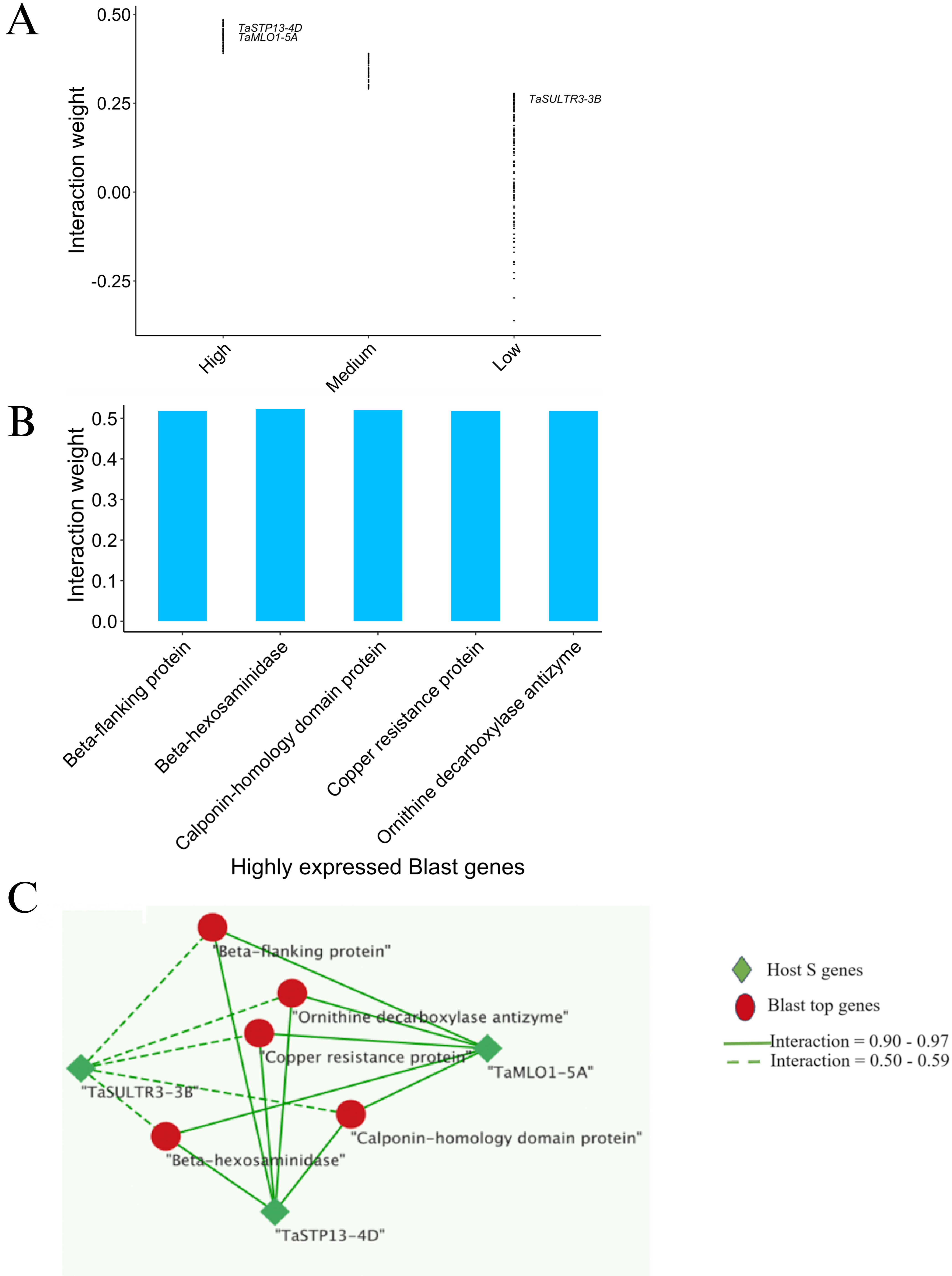

Supplementary figure 1: Network and interaction index of wheat and blast genes.
(A) Wheat genes were ranked and classified according to their interaction weights across blast genes. The interaction weight was calculated based on how many blast genes the individual wheat genes interact and their total interaction values**. (B)** Putative function of top 5 interacting genes of blast based on blast-interaction index of significantly (FDR<0.01) expressed blast genes. **(C)** Network visualizing the interactions of the highly expressed five blast genes and three S genes of wheat. Red circles denote blast genes and green shapes symbolize wheat genes. Solid lines between genes demonstrate high interaction and gapped lines denote medium/low interactions.
